# Rapid Assessment of Exercise State through Athlete's Urine Using Temperature-Dependent NIRS Technology

**DOI:** 10.1155/2020/8828213

**Published:** 2020-08-29

**Authors:** Lihe Ding, Lei-ming Yuan, Yiye Sun, Xia Zhang, Jianpeng Li, Zou Yan

**Affiliations:** ^1^School of Physical Education & Sport Science, Wenzhou Medical University, Wenzhou 325035, China; ^2^College of Electric & Electronic Engineering, Wenzhou University, Wenzhou 325035, China

## Abstract

Athletes usually take nutritional supplements and perform the specialized training to improve the performance of sport. A quick assessment of their athletic status will help to understand the current physical function of athletes' status and the effect of nutritional supplementation. Human urine, as one of the most important body indicators, is composed of many metabolites, which can provide effective monitoring information for physical conditions. In this study, temperature-dependent near-infrared spectroscopy (NIRS) technology was used to collect the spectra of athlete's urine for evaluating the feasibility of rapidly detecting the exercise state of the basketball player. To obtain the detection results accurately, several chemometrics methods including principal component analysis (PCA), variables selection method of variable importance in projection (VIP), continuous 1D wavelet transform (CWT), and partial least square-discriminant analysis (PLS-DA) were employed to develop a classifier to distinguish the physical status of athletes. The optimal classifying results were obtained by wavelet-PLS-DA classifier, whose average precision, sensitivity, and specificity are all above 0.95, and the overall accuracy of all samples is 0.97. These results demonstrate that temperature-dependent NIRS can be used to rapidly assess the physical function of athlete's status and the effect of nutritional supplementation is feasible. It can be believed that temperature-dependent NIR spectroscopy will obtain applications more widely in the future.

## 1. Introduction

To achieve a high level of athletic performance, hard trainings and reasonable dietary nutritional supplements are necessary. However, up to now, there is no rapid monitoring method to assess the exercise state of athlete and the effect of nutritional supplementation. Consequently, it usually leads to the over or under training for athlete and affects the performance and physical health of athlete [1]. Therefore, it is necessary to develop a new technology to accurately assess the exercise state and physical function of athletes. This will provide scientific training guidance and scientific information of nutritional supplements for athletes.

Body metabolites contain a variety of primary and secondary metabolites, which can reflect athlete's physical function and state and provide the most intuitive information for human health and exercise states [2]. Urine of human body is one of the most important outputs composed of many metabolites. It has been widely used for assessment of human health and disease diagnosis, and it will help to quickly understand the body's physical function and nutritional diet [3, 4]. Creatine, as an auxiliary nutritional supplement, will be metabolized and decomposed after consumption and eventually be eliminated from the body by urination [5]. Therefore, urine can be used as an analytical test to assess whether the athlete takes the sport's supplement creatine. Traditional methods of detecting the supplement creatine usually have the disadvantages of complex sample preparation, being time-consuming, and the need to use toxic and harmful reagents [6–8], which cannot meet the needs of rapid and accurate detection. Therefore, it is necessary to develop a new technology to rapid detect the status of basketball player before and after taking creatine.

Near infrared spectroscopy (NIRS) is known as a fast and nondestructive analysis technology in the wavelength range of 780–2500 nm. NIRS has been widely applied in food, agriculture, biology, and chemistry fields [9–14]. At present, many researchers have used NIRS to qualitatively and quantitatively analyze the metabolites of athletes in sports medicine fields, such as quantitative determination of glucose content in urine [15, 16], rapid detection, and analysis of trace protein in urine [17]. However, the lack of the spectral information greatly limits the application broad of NIRS [18]. To solve this problem, temperature-dependent near-infrared spectroscopy technology was proposed [19], which added the information of temperature into spectral dataset. Thus, more useful spectral information about temperature influence on analytes can be used to construct the multivariate model. As a new type of spectroscopy technology, a few applications have been applied and good analytical results were also obtained [20, 21]. Compared with traditional NIRS technology, temperature-dependent NIRS technology can effectively increase the amount of spectral information and help improve the accuracy and stability of calibration model.

Spectral data, especially temperature-dependent near-infrared (NIR) spectra data, is a kind of high-dimensional data, containing mass spectral information. Therefore, development of multivariate calibration model is usually required for dimensional reduction, denoising operation, variables selection, and vector projection [22]. The commonly used method for dimensional reduction is principal component analysis (PCA) method [23], which can be effective in extracting several principal components from high-dimensional spectra data and reducing the dimension of spectra data. In this paper, temperature-dependent NIRS with a temperature dimension that has one more dimension than that of traditional spectral dataset. Hence, suitable chemometrics method is necessary to process these high-dimensional spectral data. In addition to the PCA, the classic partial least squares discriminant analysis (PLS-DA) model was also considered to discriminate the pre- and postexcise group [24]. Additionally, another two analysis methods of variable importance in projection (VIP) [25] and continuous wavelet transform (CWT) [26, 27] were also introduced to further improve the classifying performance of the calibration model.

The main research objective of this study is to verify the feasibility of applying temperature-controlled NIRS technology to quickly discriminant analysis of pre- and postexercise states of basketball players after eating creatine. The specific goals are as follows: (1) collecting NIR data of urine samples at a series of temperature conditions; (2) making the preliminary spectral exploration with varying temperatures and sample's visualization of spectral data using PCA algorithm; (3) constructing the multivariate calibration classifiers between NIRS dataset and exercise state using PLS-DA algorithm; (4) identifying the optimal variables and enhancing the resolution of spectra using VIP and CWT algorithms; (5) comparing performances of all classifiers and identifying the best detection classifier.

## 2. Materials and Methods

### 2.1. Sample Collection and Preparation

15 male basketball players at the age range of 18 to 23 (weight range of 75 ± 5 kg) from Wenzhou Medical University were convened for this experimental trials. Participants were interviewed to obtain body information including drug intake, nutritional supplements, past medical diseases, and anthropometric data. All participants agreed to participate in the study and signed the informed consent.

Before the urine collection, all athletes are required to write down the dietary information on the day of urine collection and to drink 100 ml of water 3 hours before urine collection and underwent 48 hours without exercise. In this experiment, all athletes will have their urine collected twice before and after the specialized exercise. 15 ml urine sample was first collected before training. All athletes will take 1.5 g creatine before training and then train for 120 minutes, and the other 15 ml urine sample will be collected after 5 minutes of rest. When the collection of urine was finished, all urine samples were bottled into 15 ml centrifuge tubes and immediately refrigerated at −20°C for future use.

### 2.2. Collection Temperature-Dependent NIRS Data

The collection of temperature-dependent NIRS data from 4000 to 12000 cm^−1^ was performed on a Vertex 70 spectrometer (Bruker Optics Inc., Ettlingen, Germany). The temperature control equipment used in this study is the 2216e temperature controller (Bruker Optics Inc., Ettlingen, Germany), which can provide a precision temperature (±0.1°C). In this study, the temperature range of this experiment is from 20°C to 50°C with a step of 5°C, and the urine will be kept in the condition of 7 temperature points orderly from low to high. To increase the ratio of signal to noise and reduce the random errors, three spectra of each urine samples with scan number 64 were collected at each temperature. Finally, the NIR matrix 210 × with 2074 columns and 210 rows were obtained for analysis.

### 2.3. Multivariate Analysis Methods and Model Evaluation

#### 2.3.1. Principal Component Analysis (PCA)

Principal component analysis (PCA) is one of the most used methods in chemometrics. It is usually implemented to reduce the dimensionality of dataset and provide the score plot for visualizing the distribution of samples [28]. As a commonly used exploratory method, PCA can make the primary evaluation of similarity between samples' classes. The main principal of PCA is to convert a set of correlated variables into several linearly independent principal components (PCs) using orthogonal transformation [29]. As a result, the dimensionality of dataset is greatly reduced through PCA analysis.

#### 2.3.2. Classification Algorithm

The classical linear classification method of PLS-DA algorithm was applied in this study. The main principle of PLS-DA is to extract several latent variables (LVs), which are the linear combination of the original variables from the independent variables in the format of matrix ***X***. Then the relationship between independent variables ***X*** and dependent variables ***Y*** is established and the prediction value of each sample is obtained by this developed relationship. To achieve sample classification, class of each sample is determined according to the threshold value of class which is calculated and identified by Bayesian statistics [24]. Finally, the class of all samples is identified using PLS-DA classifier.

#### 2.3.3. CWT and VIP Methods

To enhance the resolution of spectral, remove the noise/uninformative variables, and improve the performance of calibration model, continuous 1D wavelet transform (CWT) was applied in this paper [30]. During the implement, “Sym2” wavelet filter and the scale parameter 20 were used to process the NIRS data. As known that there are many redundant and uninformative variables involved in the spectral matrix that will lead to the poor performance of the calibration model. To solve this issue, variable selection was commonly used to identify the optimal variables prior to establishing the multivariate model. In this study, variable importance in projection (VIP) was considered to select the optimal variables. During the processing stage of VIP, V value that reflects the contribution of each variable was calculated based on the PLS regression to identify the optimal variable [31]. Finally, those optimal variables with V value larger than 1 were eventually selected.

#### 2.3.4. Model's Evaluation

In this study, four evaluation parameters, namely, sensitivity, specificity, precision, and accuracy, are used to evaluate the performance of the classification model. They can accurately and objectively evaluate the performance of PLS-DA classification model. A classification model with good performance should have the high value of sensitivity, specificity, precision, and accuracy [24, 32]. All algorithms and calculations are carried out in the MATLAB 2015b environment (The Math Works, Natick, USA).

## 3. Results and Discussion

### 3.1. Spectral Profile Analysis

**Figure 1:**
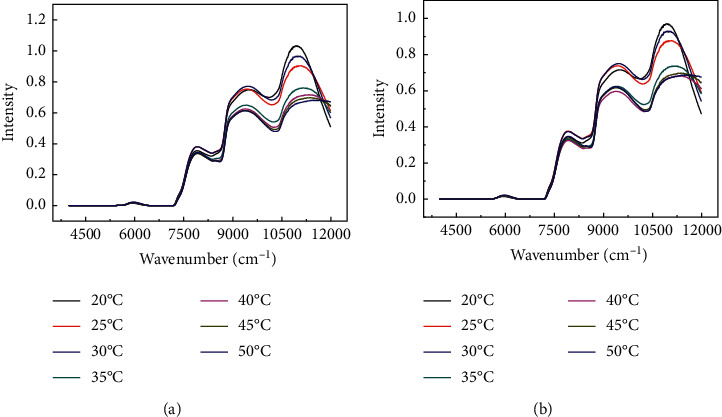
Average spectrum of basketball player's urine with different temperature measurements. (a) Before exercise; (b) after exercise.

In this study, temperature-dependent NIRS data of basketball players' urine were collected in temperature ranges from 20 to 50 degrees Celsius in steps of 5. The corresponding spectral profiles are shown in Figure 1, where the average spectral curves of the seven temperatures are collected before and after the basketball players' exercise. It can be found that there are several obvious absorption peaks at 7800 cm^−1^, 9300 cm^−1^, and 10800 cm^−1^, which may be caused by function groups of C-H, H-O, N-H, and C-O band in water, glucose, and protein from the urine [2]. Obviously, there is no significant difference in spectral profiles between before and after exercise of basketball player by eye-naked judgement. On the consideration of spectral intensity, it can be found that the spectral intensity generally shows a downward trend with increasing temperature except for the spectra of 30°C. Besides, with increasing temperature of urine sample, the spectral intensity at 11250 cm^−1^ is bizarre and decreases with big jump at low temperature (20°C, 25°C, and 30°C) but small slope at high. The possible reason is that the substances in urine from 25°C to 30°C have been changed. On the other hand, although the spectral intensity decreases with the temperature, it is difficult to fit using linear functions. Based on the above analysis, it can be found that it is hard to directly distinguish the samples before and after training only by observing the intensity of spectral curve. Therefore, multivariate calibration model will be applied in the subsequent analysis to further analyze the spectral dataset.

### 3.2. PCA of Temperature-Dependent NIRS Data

**Figure 2:**
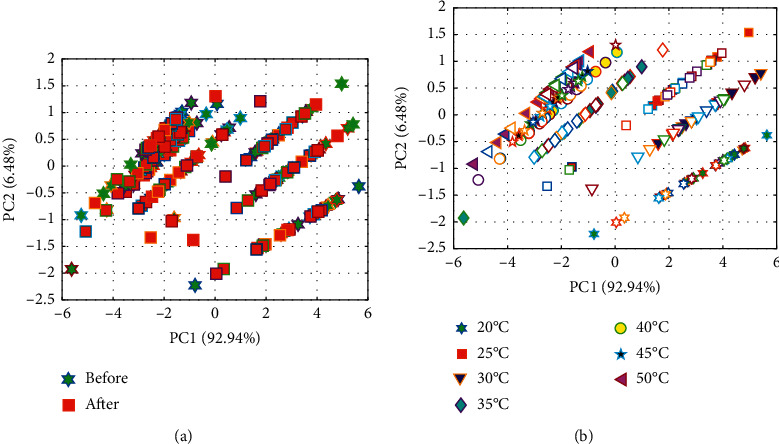
Two-dimensional PCA and 2D-PCA analysis maps for the two classes of urine samples. (a) PCA plots of 210 urine spectral samples at 20°C. (b) PCA plots of urine samples at different temperature.

Prior to the calibrating analysis, it is recommended to explore the structure of spectral dataset. In this study, the effective statistical method of PCA was used to explore and visualize the space distribution of samples by extracting several new principal components from high-dimensional dataset. PCA was firstly performed on the temperature-dependent NIRS dataset to plot the score scatter of samples and observe the sample's distribution.  Figure 2(a) shows the PCA score plots of 210 urine spectral samples collected from before and after training. It is clear that PC1 and PC2 account for 92.94% and 6.48% contributions to the original spectra, respectively, and the total cumulative variance contributes close to 99.42%. However, the samples from before and after training were still overlapped in these two-dimensional space, which indicated that the urine samples from before and after training were difficult to separate based on first two PCs. To further explore the distribution of samples, 210 samples coming from different temperature of 20°C–50°C were considered.

Taking a close observation on Figure 2(b), it shows the distribution of samples with some linear relationship at the same temperature. In addition, with rising of the temperature of urine sample, samples are distributed from the lower right to the upper left except for 30°C. Moreover, there is no sample overlapped among different temperatures. These indicate that the temperature will significantly affect the NIRS response signals of urine samples. But PCA is an unsupervised method and cannot accurately distinguish the categories of urine samples. The possible reason is that the change of substances composition in urine before and after exercise is small, and the unsupervised principal component analysis method cannot fully exhibit this change only using several PCs. As a result, PCA failed to accurately distinguish these samples that belong to two classes. To solve this issue, more information needed to be mined from NIRS data, and the classic PLS-DA classification model will be performed to construct the classification model.

### 3.3. PLS-DA Model Based on Full and CWT Variables

Based on the above analysis, the unsupervised PCA method cannot directly distinguish urine samples from the class of before and after training. Therefore the multivariate modeling method of PLS-DA was used to create the classification model. Prior to establishing the PLS-DA model, 210 samples were randomly divided into the calibration set and prediction set with the ratio of 2 : 1. Then the classification model was established based on the calibration set, and the number of latent variables (LVs) involved in PLS-DA model was optimized using 10-fold cross-validation and was determined at the lowest root mean square error of cross-validation (RMSECV). The specifically calculated results are shown in Table 1.

First of all, PLS-DA classification model was established based on the full variables of raw spectra to distinguish the urine sample. In Table 1, it can be seen that the values of sensitivity, specificity, and precision in calibration set are all 100%. However, the result of validation and prediction set is lower than that in the calibration set. For urine samples from the before-training group, the precision, sensitivity, and specificity in prediction set are 0.78, 0.82, and 0.71, respectively; meanwhile, the after-training group is 0.76, 0.71, and 0.82, and the overall classification accuracy of all samples is 0.77. This result indicates that it is possible to rapidly detect the athletic state of athlete using NIRS technology. To further improve the performance of classification model, continuous 1D wavelet transform was proposed to transform the temperature-dependent NIRS dataset, and this would help improve the resolution of spectra. Then the PLS-DA classification model was built as in the previous step, and its performance was compared with the full-variables-based PLS-DA model. In Table 1, it can be found that the precision, sensitivity, and specificity of class 1 (labelled “before training”) and class 2 (labelled “after training”) in prediction set are 0.95, 1.00, and 0.94 and 1.00, 0.94, and 1.00, and the overall accuracy reached 0.97. Obviously, the performance of PLS-DA classification model has been significantly improved compared to that of full-variables-based PLS-DA model. The result shows that the continuous 1D wavelet transform (CWT) is an effective way to enhance the resolution and further improve the accuracy of model.

### 3.4. PLS-DA Model Based on Optimal Variables

Although a high classification accuracy has been obtained by the CWT-PLS-DA model, the calculating process of these models is complex due to too many variables involved in the calculation model. Moreover, it is known that there are many irrelevant, collinear, and redundant variables in spectral data which will lead to poor performance of classification model. Therefore, it is necessary to hunt suitable variable selection algorithms to identify a few important variables before establishing PLS-DA classification model [33]. Employments of variable selections not only help reduce the dimensionality of spectral data and the complexity of computation but also improve the accuracy and robustness of the model. For this reason, classic variable selection algorithm VIP was applied on raw spectra data and the spectral data processed by continuous 1D wavelet transform algorithm.

**Figure 3:**
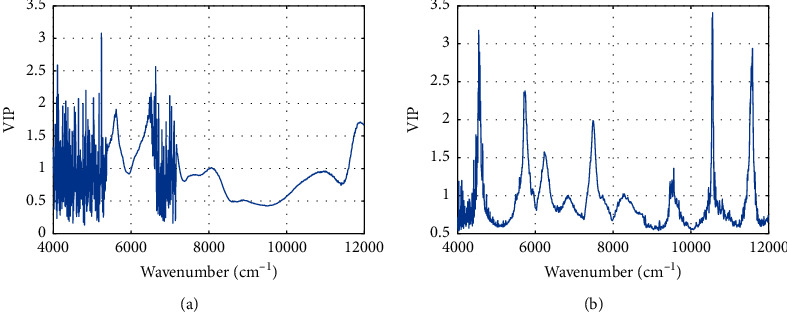
Optimal variables selected by VIP from raw spectrum (a) and wavelet transformed spectrum (b).

Results of variables selection through VIP are shown in Figure 3. According to the criterion of VIP algorithm, those variables will be selected as the optimal variables whose VIP is larger than 1. Therefore, variables above the bold black line in Figure 3 are selected as optimal variables. Specifically, 604 and 483 feature variables were identified from raw spectrum and wavelet-transformed spectrum. Compared with 2074 full variables, the number of variables was decreased by 70.87% and 76.71%, and the dimension of the spectra had been greatly decreased. When the variables selection was completed, those feature variables that contained the most useful information related to urine of basketball player were used to construct the PLS-DA classification model and the corresponding results were shown in Table 1. It can be seen that the performance of VIP-PLS-DA model in terms of the model precision (0.78 versus 0.88 and 0.76 versus 0.86), sensitivity (0.82 versus 0.90 and 0.71 versus 0.83), and specificity (0.71 versus 0.83 and 0.82 versus 0.90) has been improved when compared with FULL-PLS-DA model. The possible reason for this improvement is that the unrelated variables may be deleted by VIP algorithm and the model becomes more stable and accurate. However, the opposite result was obtained when the wavelet-VIP-PLS-DA model was considered compared with FULL-PLS-DA model.

Specifically, there are no significant differences between performances of these PLS-DA models in class 2, but the performance for class 1 is worse than FULL-PLS-DA model. It demonstrates that although the continuous 1D wavelet can improve the performance of PLS-DA, the subsequent variable selection may not be suitable when the spectral data was transformed by continuous 1D wavelet. Therefore, only CWT pretreatment is the better way to analyze NIRS data. In addition, it can be found that there are many noise variables in the range of 4000–5400 cm^−1^ and 6500–7200 cm^−1^ in Figure 3(a), which are contrary to the characteristic of continuous spectrum. To eliminate the effect of those noise variables, we also performed the calculation only using the variables from the range of 11600–12000 cm^−1^ and 5400 cm^−1^–6500 cm^−1^, and the overall accuracy is about 0.85, which is consistent with the VIP-PLS-DA model. This means those unstable variables in the range of 4000–5400 cm^−1^ and 6500–7200 cm^−1^ do not work or even negatively affect the PLS-DA classification model.

When all classification models, including FULL-PLS-DA, VIP-PLS-DA, wavelet-PLS-DA, and wavelet-VIP-PLS-DA, are considered and compared, the best classification model is wavelet-PLS-DA, whose the overall accuracy reaches 0.97, and the better one is VIP-PLS-DA model. The worst one is the wavelet-VIP-PLS-DA model with accuracy of 0.77. These results demonstrate that it is feasible to use multivariate calibration model and NIRS data to determine the exercise status of athlete. In this primary study, there is still a lot of work that needs to be further improved and supplemented, such as more exercise types, more nutritional supplements, more reasonable experimental designs, and more effective analysis methods.

## 4. Conclusion

In this study, the urine samples of basketball players were collected from before- and after-training groups and were measured using NIRS technology, coupled with the newly proposed temperature-dependent approach in the temperature range of 20°C to 50°C with step of 5°C to collect the NIRS data. To distinguish the exercise state of athletes, the classic linear classification method PLS-DA was established based on the processed variables that were preprocessed by CWT, VIP, and their combinations. Comprehensively, comparing performances of all PLS-DA models, CWT-PLS-DA has the best performance whose average precision, sensitivity, and specificity in prediction set are 0.98, 0.97, and 0.97, respectively. The result indicates that temperature-dependent NIRS is a potential technique to accurately assess the exercise status of athletes and will help optimize the amount of training and nutritional supplements for athletes in the further.

## Figures and Tables

**Table 1:** Classification results of PLS-DA model based on raw variables, optimal variables selected from raw spectra, and optimal variables from CWT spectra.

Model	Methods	LVs^1^	Class	Calibration			Validation			Prediction		
				Pre^2^	Sen^3^	Spe^4^	Pre	Sen	Spe	Pre	Sen	Spe
PLS-DA	—	7	1	1.00	1.00	1.00	0.71	0.83	0.69	0.78	0.82	0.71
			2	1.00	1.00	1.00	0.82	0.69	0.83	0.76	0.71	0.82
	VIP	5	1	1.00	1.00	1.00	0.88	0.91	0.89	0.88	0.90	0.83
			2	1.00	1.00	1.00	0.92	0.89	0.91	0.86	0.83	0.90
	Wavelet	8	1	1.00	1.00	1.00	0.99	1.00	0.99	0.95	1.00	0.94
			2	1.00	1.00	1.00	1.00	0.99	1.00	1.00	0.94	1.00
	Wavelet-VIP	9	1	1.00	1.00	1.00	0.86	0.86	0.82	0.66	0.81	0.75
			2	1.00	1.00	1.00	0.82	0.82	0.86	0.87	0.75	0.81

Notes: 1: latent variables; 2: precision; 3: sensitivity; 4: specificity.

## Data Availability

All the data supporting the current findings reported in this manuscript are available from the corresponding author upon request.

## References

[1] Meeusen R., Duclos M., Foster C. (2013). European college of sport science; American college of sports medicine. Prevention, diagnosis, and treatment of the overtraining syndrome: joint consensus statement of the European college of sport science and the American college of sports medicine. *Medicine & Science in Sports & Exercise*.

[2] Moreira L. P., Silveira L., Pacheco M. T. T., Rocco D. D. F. M. (2018). Detecting urine metabolites related to training performance in swimming athletes by means of Raman spectroscopy and principal component analysis. *Journal of Photochemistry and Photobiology B: Biology*.

[3] Han W. K., Waikar S. S., Johnson A. (2008). Urinary biomarkers in the early diagnosis of acute kidney injury. *Kidney International*.

[4] Smith S. D., Wheeler M. A., Plescia J., Colberg J. W., Weiss R. M., Altieri D. C. (2001). Urine detection of survivin and diagnosis of bladder cancer. *JAMA*.

[5] Guinovart T., Hernández-Alonso D., Adriaenssens L. (2017). Characterization of a new ionophore-based ion-selective electrode for the potentiometric determination of creatinine in urine. *Biosensors and Bioelectronics*.

[6] Sununta S., Rattanarat P., Chailapakul O., Praphairaksit N. (2018). Microfluidic paper-based analytical devices for determination of creatinine in urine samples. *Analytical Sciences*.

[7] Erenas M. M., Ortiz-Gómez I., de Orbe-Payá I. (2019). Ionophore-based optical sensor for urine creatinine determination. *ACS Sensors*.

[8] Jurdáková H., Górová R., Addová G., Šalingová A., Ostrovský I. (2018). FIA-MS/MS determination of creatinine in urine samples undergoing butylation. *Analytical Biochemistry*.

[9] Pasquini C. (2018). Near infrared spectroscopy: a mature analytical technique with new perspectives—a review. *Analytica Chimica Acta*.

[10] Wang L., Sun D.-W., Pu H., Cheng J.-H. (2017). Quality analysis, classification, and authentication of liquid foods by near-infrared spectroscopy: a review of recent research developments. *Critical Reviews in Food Science and Nutrition*.

[11] Grassi B., Quaresima V. (2016). Near-infrared spectroscopy and skeletal muscle oxidative functionin vivoin health and disease: a review from an exercise physiology perspective. *Journal of Biomedical Optics*.

[12] Yuan L.-M., Mao F., Chen X., Li L., Huang G. (2020). Non-invasive measurements of “Yunhe” pears by vis-NIRS technology coupled with deviation fusion modeling approach. *Postharvest Biology and Technology*.

[13] Zareef M., Kutsanedzie F. Y. H., Agyekum A. A. (2020). An overview on the applications of typical non-linear algorithms coupled with nir spectroscopy in food analysis. *Food Engineering Reviews*.

[14] Sheng R., Cheng W., Li H., Ali S., Akomeah Agyekum A., Chen Q. (2019). Model development for soluble solids and lycopene contents of cherry tomato at different temperatures using near-infrared spectroscopy. *Postharvest Biology and Technology*.

[15] Kim H., Allen D. G. (2016). Using digital filters to obtain accurate trended urine glucose levels from toilet-deployable near-infrared spectrometers. *Journal of Analytical & Bioanalytical Techniques*.

[16] Yamamoto N., Kawashima N., Kitazaki T. (2018). Ultrasonic standing wave preparation of a liquid cell for glucose measurements in urine by midinfrared spectroscopy and potential application to smart toilets. *Journal of Biomedical Optics*.

[17] Raghavachari R. (2019). Biomedical applications of near-infrared spectroscopy. *Near-Infrared Applications in Biotechnology*.

[18] Liu K., Chen X., Li L., Chen H., Ruan X., Liu W. (2015). A consensus successive projections algorithm—multiple linear regression method for analyzing near infrared spectra. *Analytica Chimica Acta*.

[19] Sun Y., Cui X., Cai W., Shao X. (2020). Understanding the complexity of the structures in alcohol solutions by temperature-dependent near-infrared spectroscopy. *Spectrochimica Acta Part A: Molecular and Biomolecular Spectroscopy*.

[20] Liu X.-W., Cui X.-Y., Yu X.-M., Cai W.-S., Shao X.-G. (2017). Understanding the thermal stability of human serum proteins with the related near-infrared spectral variables selected by Monte Carlo-uninformative variable elimination. *Chinese Chemical Letters*.

[21] Cui X., Yu X., Cai W., Shao X. (2019). Water as a probe for serum-based diagnosis by temperature-dependent near-infrared spectroscopy. *Talanta*.

[22] Xiaobo Z., Jiewen Z., Povey M. J. W., Holmes M., Hanpin M. (2010). Variables selection methods in near-infrared spectroscopy. *Analytica Chimica Acta*.

[23] Wold S., Esbensen K., Geladi P. (1987). Principal component analysis. *Chemometrics and Intelligent Laboratory Systems*.

[24] Ballabio D., Consonni V. (2013). Classification tools in chemistry. Part 1: linear models. PLS-DA. *Analytical Methods*.

[25] Farrés M., Platikanov S., Tsakovski S., Tauler R. (2015). Comparison of the variable importance in projection (VIP) and of the selectivity ratio (SR) methods for variable selection and interpretation. *Journal of Chemometrics*.

[26] Watkins L. R. (2012). Review of fringe pattern phase recovery using the 1-D and 2-D continuous wavelet transforms. *Optics and Lasers in Engineering*.

[27] Ye P., Ji G., Yuan L.-M. (2019). A sparse classification based on a linear regression method for spectral recognition. *Applied Sciences*.

[28] Chen X., Ding H., Yuan L.-M., Cai J.-R., Chen X., Lin Y. (2018). New approach of simultaneous, multi-perspective imaging for quantitative assessment of the compactness of grape bunches. *Australian Journal of Grape and Wine Research*.

[29] Ringnér M. (2008). What is principal component analysis?. *Nature Biotechnology*.

[30] Cui X., Liu X., Yu X., Cai W., Shao X. (2017). Water can be a probe for sensing glucose in aqueous solutions by temperature dependent near infrared spectra. *Analytica Chimica Acta*.

[31] Nie M., Meng L., Chen X. (2019). Tuning parameter identification for variable selection algorithm using the sum of ranking differences algorithm. *Journal of Chemometrics*.

[32] Chen X., Xu Y., Meng L. (2020). Non-parametric partial least squares-discriminant analysis model based on sum of ranking difference algorithm for tea grade identification using electronic tongue data. *Sensors and Actuators B: Chemical*.

[33] Yuan L.-M., Cai J.-R., Sun L., Han E., Ernest T. (2016). Nondestructive measurement of soluble solids content in apples by a portable fruit analyzer. *Food Analytical Methods*.

